# Social inequalities in the burden of care: a dyadic analysis in the caregiving partners of persons with a physical disability

**DOI:** 10.1186/s12939-019-1112-1

**Published:** 2019-12-31

**Authors:** Hannah Tough, Martin W. G. Brinkhof, Johannes Siegrist, Christine Fekete, for the SwiSCI Study Group

**Affiliations:** 1 Swiss Paraplegic Research, Guido A. Zäch Strasse 4, 6207 Nottwil, Switzerland; 2 Department of Health Sciences and Medicine, University of Lucerne, Frohburgstrasse 3, 6002 Lucerne, Switzerland; 3 Senior Professorship ‘Work Stress Research’, Faculty of Medicine, University of Düsseldorf, Life-Science-Center, Merowingerplatz 1A, 40225 Düsseldorf, Germany

**Keywords:** Caregiver burden, Dyadic analysis, Inequalities, Mediation, Socio-economic position, Spinal cord injury, Stress process model of caregiving

## Abstract

**Background:**

Socioeconomic position (SEP) is an important contextual factor in the Stress Process Model of caregiving. However, the basic assumption that low SEP is associated with greater caregiver burden has so far lacked empirical support. The objective of this study was to investigate social inequalities in the caregiver burden among caregiving partners of persons with a physical disability, i.e., spinal cord injury (SCI), applying a dyadic approach. More specifically, we investigated 1) the association of the caregivers’ SEP with caregiver burden (‘*actor effect’*); 2) the association of the care-receivers’ SEP with caregiver burden (‘*partner effect’*), and 3) potential mediators of the association between SEP and caregiver burden.

**Methods:**

Cross-sectional survey data from 118 couples of persons with SCI and their partners living in Switzerland was used. We firstly employed logistic regression to investigate the actor and partner effects of SEP on objective (hours of caregiving) and subjective caregiver burden (Zarit Burden Interview). We additionally used structural equation modelling to explore whether unfulfilled support needs, psychosocial resources and the care-receivers health status mediated the association between SEP and caregiver burden. SEP was operationalized by household income, education, subjective social position, financial strain and home ownership.

**Results:**

We observed a consistent trend towards higher objective and subjective burden in lower SEP groups. Caregivers with higher subjective social positon and home ownership indicated lower subjective burden, and caregivers with higher education and absence of financial strain reported lower objective burden. Further evidence suggested a partner effect of SEP on caregiver burden, whereby objective caregiver burden was reduced in couples where the care-receiver had a higher educational level. The negative association between SEP and subjective burden was partially mediated by the unfulfilled support needs and deprived psychological resources of the caregiver, and the poor health status of the care-receiver. Similar mediation effects were not supported for objective burden.

**Conclusions:**

Our study, in the context of SCI, provides support for the contextual role of SEP in the Stress Process Model of caregiving. To reduce subjective caregiver burden, policy programs may target the strengthening of psychosocial resources, or the improvement of access to support services for caregivers with low SEP.

## Background

The demand for informal care is rising, largely due to an ageing population, and a subsequent increase in persons living with chronic conditions and functional limitations [1, 2]. Informal care is defined as non-professional care provided for persons with long-term care needs by family members, friends, neighbours or other individuals. In Switzerland, roughly 21% of the population are involved in informal caregiving [3] and several contemporary health initiatives seek to reduce the financial and psychological burden of informal caregiving, with the aim to ultimately improve caregivers’ health [4]. To inform and evaluate the efficacy of such programs, the caregivers’ experience of objective and subjective burden needs to be systematically monitored and documented. While the subjective burden refers to the psychological or emotional impact which caregiving responsibilities have on the caregiver [5], the objective burden refers to the time burden and the number of activities for which the care-receiver requires assistance.

Socioeconomic position (SEP) may play a key role in determining the burden of care. While the current literature generally supports the notion that caregivers in lower SEP incur higher objective burden [6–8], few studies have explored the impact of SEP on subjective burden [9, 10]. Moreover, the theoretical pathways which link SEP to objective and subjective burden are generally not investigated. According to the Stress Process Model of caregiving, informal caregivers with lower SEP are expected to be at higher risk of caregiver burden due to their limited access to stress-buffering resources [11]. Material, psychosocial and behavioral stress-buffering resources may be more accessible to caregivers with higher SEP, potentially enabling these caregivers to better cope with their caring role, and therefore limiting their burden of care.

To facilitate policy that aims to reduce social inequalities in caregiver burden, it is important to explore possible mediating factors and underlying processes in the association of SEP with caregiver burden [12]. Previous research has identified several potential mediating factors which are unequally distributed within society, including psychosocial resources, such as caregivers’ coping strategies, level of self-efficacy, and satisfaction with social connections [13–17], as well as material resources, such as access to professional support, healthcare information and healthcare services [18]. Poor access to stress-buffering resources may not be the only pathway through which SEP impacts upon caregiver burden. Evidence suggests that the prevalence of disability, and therefore the need for informal care, is highest in those with the lowest SEP [6, 7, 19–21]. Not only are disabling conditions more prevalent in those with the lowest SEP, but those already affected by a disability are more prone to secondary health conditions and comorbidities if they are socially disadvantaged [22–24]. For example, one study in the context of spinal cord injury (SCI) has shown that individuals reporting financial strain also suffered from poorer physical and mental functioning [22].

**Fig. 1:**
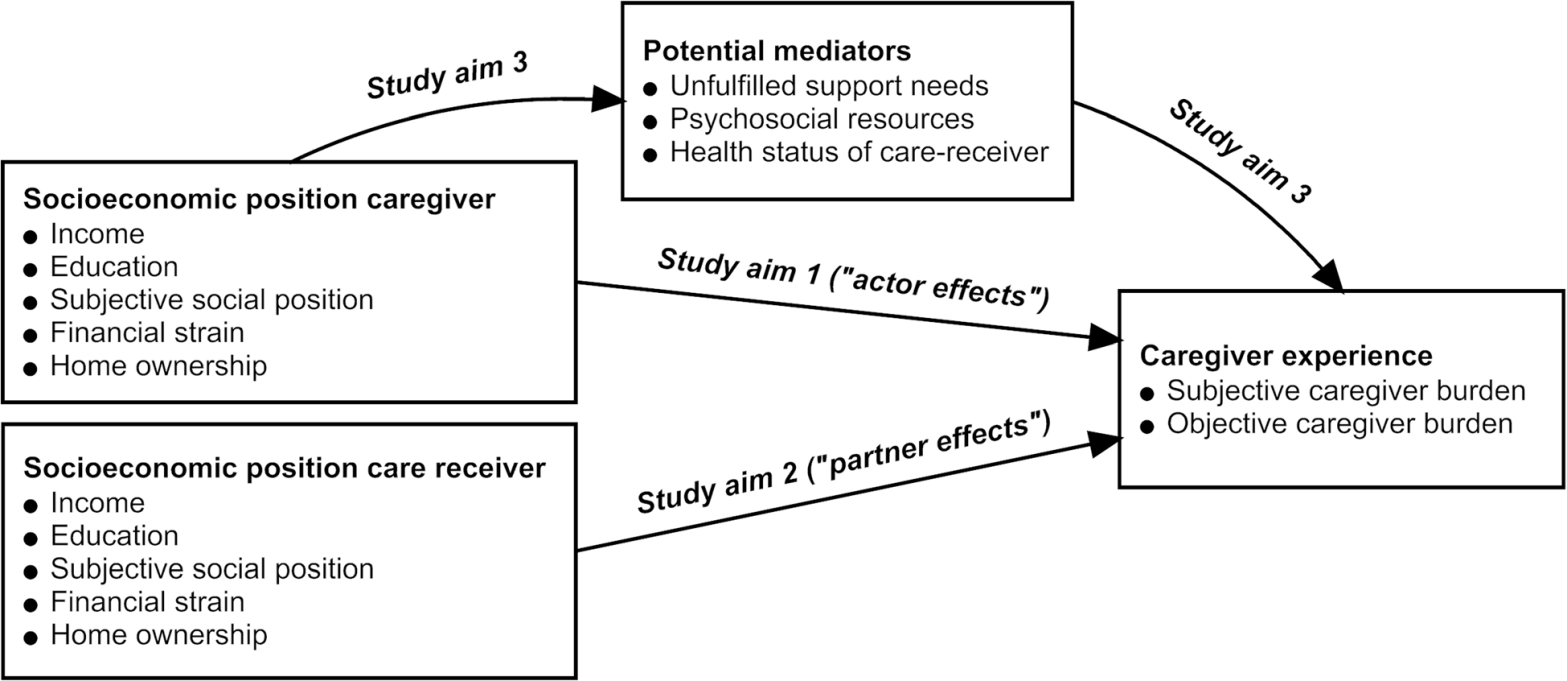
Overview of the study aims

We investigated social inequalities in the caregiver burden in the context of SCI in Switzerland. An SCI is a chronic traumatic or non-traumatic damage to the spinal cord that leads to a total or partial loss of sensation and movement below the lesion level and oftentimes leads to major physical disability [25]. Although prevalence is rather low - with worldwide estimates ranging from 280 to 2500 cases per million population – SCI has an extensive impact on functioning, health and well-being, and results in major individual and societal costs [25]. This disabling condition offers an informative case in point, as it often leads to dependency on informal care and the need for support in numerous activities of daily living [25, 26]. Importantly, our analysis set in the Swiss context presents an informative case for the development of policy programmes in high income countries that seek to take account for social inequalities in the broader context of caregiving. Characteristically, social inequalities in health are also manifest in Switzerland, despite a high-level gross domestic product (GDP) per capita, a highly developed health care system, extended social security policies and moderate levels of wealth inequality [21, 27, 28]. To depict the SEP of persons with SCI and their caregiving partners, we utilized a comprehensive set of indicators, including education, household income and home ownership, as well as indicators that may affect people’s daily lives more directly, such as financial strain and subjective social position. In light of the substantial research gaps identified in caregiver research, the overall objective of this study is to investigate social inequalities in the caregiver burden applying a dyadic perspective, including the SEP of both the caregiver (‘actor’) and the care-receiver (‘partner’). More specifically, we investigated 1) whether the SEP of the caregiver is associated with objective and subjective caregiver burden (‘actor effects’), 2) whether the SEP of the care-receiver (i.e., person with SCI) is associated with objective and subjective caregiver burden (‘partner effects’), and 3) whether the association between the SEP and caregiver burden is mediated by unfulfilled support needs, psychosocial resources and the care-receivers health status (Fig. 1). Based on evidence from other study populations, we hypothesize that lower SEP of the caregiver is associated with higher caregiver burden, 2) that lower SEP of the care-receiver is associated with higher caregiver burden, and 3) that the potential association between low SEP and higher caregiver burden is mediated by unfulfilled support needs, low psychosocial resources and poor health status of the care-receiver.

## Methods

### Sampling frame and participants

Data for this analysis came from the pro-WELL study [29]. Pro-WELL participants were recruited from the community survey of the Swiss Spinal Cord Injury Cohort Study (SwiSCI), which was performed between September 2011 and March 2013. The SwiSCI sampling frame included a representative sample of 1922 persons with traumatic or non-traumatic SCI living in Switzerland, aged 16 years or older [30, 31]. The following eligibility criterion were applied for pro-WELL: aged 30–65 years, fluent in German or French, living in a stable partnership, having a partner that is involved in caregiving tasks and was willing to participate in the study, as only couples were included. In total, 676 persons with SCI from the sampling frame were deemed eligible, and 133 persons with SCI and their partners were recruited at baseline (total *n* = 266; response rate 19.7%). A comprehensive non-response analysis demonstrated good representation of the source population, with insignificant selection bias regarding sociodemographic and lesion characteristics [29]. In this analysis, only the partners of persons with SCI who indicated supporting their partner in activities of daily living were included (*n* = 118).

### Study design

Pro-WELL is a longitudinal community survey with three measurement waves (baseline; month 6; month 12). Data were collected using computer-assisted telephone interviews and questionnaires (paper-pencil or online). Given that the SEP and the subjective caregiver burden were only assessed at baseline, only cross-sectional data from the baseline assessment that was carried out between May 2015 and January 2016 were used in this analysis. The study protocol and all measures were approved by the Ethical Committee of Northwest and Central Switzerland (document EKNZ 2014–285). Pro-WELL strictly observed all regulations concerning informed consent and data protection and conforms to the Helsinki Declaration.

### Measures

#### Predictor: socioeconomic position

Education, household income, financial strain, subjective social position and home ownership were used to operationalize the SEP of caregiving partners and persons with SCI. Education was assessed according to the International Standard Classification of Education as total years of formal education, combining school and vocational training [32]. For the persons with SCI, potential re-training after the onset of SCI was not added to the total years of education. We used net-equivalent household income to assess the income situation of participants. Net-equivalent household income represents the total of disposable income of the household, weighted by the total number of adults and children living in the household. OECD criteria were applied for the weighting (i.e., 1.0 for the first adult; 0.5 for each additional adult; 0.3 for each child in the household) [33]. Financial strain was measured with a 5-point Likert scaled item asking participants how they get along with their available financial resources. The available response options were: financial resources are very scarce, scarce, just last, last well, or last very well. For analysis, the variable was dichotomized into ‘financial strain’ (including the responses ‘very scarce’ and ‘scarce’) and ‘no financial strain’ (including the responses ‘just last’, ‘last well’, and ‘last very well’). The MacArthur Scale of Subjective Social Status was used to capture the subjective evaluation of one’s position in society, represented by a 10-rung ladder [34]. A single item was used to assess home ownership (yes vs. no). Information on total years of education, net-equivalent household income and subjective social position (scale 0–10) was used continuously for analysis.

#### Outcome: caregiver burden

Objective caregiver burden was measured by the daily hours of caregiving. For multivariable analysis those performing at least 3 h of daily caregiving were categorised as having high objective burden [6]. Subjective caregiver burden assesses the personal feelings of strain resulting from the caregiving role measured with the Zarit Burden Interview short form (ZBI-S). The twelve items were rated on a 5-point scale, measuring the frequency of feelings of burden (never, rarely, sometimes, frequently, nearly always). We calculated a sum score ranging from 0 to 48 [35, 36] and Cronbach’s alpha across the twelve items was 0.88, indicating good internal consistency. As a rule of thumb, Cronbach’s alpha was interpreted as follows: ≥0.9 excellent; ≥0.8 to < 0.9 good; ≥0.7 to < 0.8 acceptable; below 0.7 unacceptable internal consistency [37]. For multivariable analysis a cut off score of 12 or higher was used to classify caregivers into a higher and a lower burden group [38, 39].

#### Potential mediators

##### Unfulfilled support needs

One item on unfulfilled support needs was used as a proxy to measure access to, and satisfaction with available professional caregiving support. The item was added as a dichotomous variable into mediation analysis.

##### Psychosocial resources

We use the partner relationship quality and self-efficacy as indicators for psychosocial resources of the caregiver, and feelings of loneliness as an indication for lack of social resources. Quality of partner relationship was assessed using eight items from the social support and depth subscales of the Quality of Relationship Inventory (QRI) which were rated on a 4-point Likert scale, resulting in a sum score ranging from 0 to 24 and indicating higher relationship quality with higher ratings [40]. The Cronbach’s alpha across the eight items was 0.82, demonstrating good internal consistency in our sample [37]. Three items from the Revised UCLA loneliness scale were used to capture subjective feelings of loneliness, with a score ranging from 0 to 6, with higher scores indicating more loneliness [41]. This scale has recently been validated in SCI [42]. General self-efficacy, which describes the general confidence in one’s own abilities to overcome difficulties, was assessed using a modified version of the General Self-Efficacy Scale (GSES) consisting of five items. Participants were asked to rate different statements, as for example ‘I can find a solution for every problem’ or ‘I know how to act in an unexpected situation’, on a four-point Likert scale ranging from 1 ‘not true’ to 4 ‘exactly true’. A sum score ranging from 5 to 20 was built, with higher scores indicating higher self-efficacy [43]. The Cronbach’s alpha across the five items indicated good internal consistency (0.85) [37].

##### Health status of care-receiver

Ten items of the Self-Administered Comorbidity Questionnaire (SCQ) were used to assess the number of comorbidities which the care-receiver reported on a dichotomous level (comorbidity present vs. absent) [44]. A sum score over the ten items was built for analysis. Nine selected items from the SCI Secondary Conditions Scale (SCI-SCS) were used to assess conditions which are commonly encountered by persons with SCI, such as pressure ulcers, urinary tract infections and bladder and bowel dysfunction. The assessment referred to the past 4 weeks and was made on a 4-point Likert scale, with ordinal levels ‘not existing or insignificant’; ‘mild or infrequent’; ‘moderate or occasional’; and ‘significant or chronic’ [45]. We calculated a sum score over the nine items (range 0–24) for analysis, the Cronbach’s alpha over the items was 0.70, indicating acceptable internal consistency [37].

#### Potential confounders

The selection of confounders was informed by directed acyclic graphs (DAGs; www.dagitty.net). The visualization of DAGs facilitates the identification of ‘true’ confounders by displaying complex interrelations between predictors, outcomes and potential candidate confounders [46]. Once literature-based confounders were identified, the theoretical DAG was validated by bivariate analysis using pro-WELL data. Thereby, age, sex, employment status of the caregiver and lesion characteristics of the care-receiver were identified as true confounders to be included in the analysis of study aim 1 and 2.

### Statistical analysis

Analysis was conducted using Stata version 14.2 for Windows (College Station, TX, USA). We first described the distribution of SEP, caregiver burden and potential mediating and confounding variables. Next, we computed bivariate within-person and within-dyad correlations of SEP in order to assess correlations between constructs but also correlations of SEP variables between the two members of a dyad. Descriptive analysis was performed with crude data, excluding all cases with missing values. To account for item non-response in predictor and control variables, the multivariable analysis for study aim 1 and 2 used multiply imputed data that were derived by multiple imputation using chained equations (MICE) [47]. Outcome variables were not imputed. Selection bias due to unit non-response has been shown to be negligible [29].

Logistic regression was used to explore actor (*study aim 1*) and partner effects (*study aim 2*) of SEP on the objective and subjective caregiver burden. We use the Actor Partner Interdependence Model (APIM) to inform our dyadic analysis [48, 49]. Indicators of SEP were entered individually into all models in order to avoid bias arising from mutual adjustment [50]. We report odds ratios (OR), 95% confidence intervals (CI) and *p*-values from Equal Fraction Missing Information (FMI) tests.

*Study aim 3* was addressed using structural equation modelling in order to include potential mediators in the association between SEP and caregiver burden. In order to evaluate the overall and accumulative effect of SEP, a latent construct was created which used education, income, subjective social position and financial strain as indicators. The latent construct was created using confirmatory factor analysis, with all indicators having satisfactory factor loadings above 0.5 [51]. Given that the factor loading for ‘home ownership’ was below 0.5, this variable was excluded from the model. The structural equation models were specified according to the study aims outlined in Fig. 1. If a potential mediator was not significantly associated with both the predictor (SEP) and the outcome (caregiver burden) there was no empirical evidence for mediation, and the variable was dropped from analysis. The final models included the mediators unfilled support needs (wish for more support), psychosocial resources (partner relationship quality; loneliness; self-efficacy), and the health status of the care-receiver (comorbidities; secondary conditions of the person with SCI). Bias-corrected and accelerated bootstrapping with 5000 replications with replacements was computed in order to deal with sample size and non-normality issues. This also enabled the estimation of asymmetrical CIs for the indirect effects in mediation analysis and for multiple mediation models [52]. Adequate model fit was assessed by a non-significant X^2^ test (vulnerable to sample size), a comparative fit index (CFI) > 0.95, and the root mean square error of approximation (RMSEA) < 0.06. We report standardized regression coefficients and 95% CIs. Our sample sufficed the minimal recommended sample size and supported the estimation of one latent construct as indicated by the ratio of the estimated parameters to the observed variables [51]. SEM analyses were conducted on non-imputed data using full information maximum likelihood (FIML) estimation, which adequately accounts for missing data [53]. Proportion of mediated effects was calculated using the *estat teffects* command in STATA, subsequently the indirect effect of SEP on caregiver experience was divided by the total effect. The proportion of explained variance in caregiver burden was reported with the R-squared statistic.

## Results

Basic characteristics of the pro-WELL sample are displayed in Table 1. The majority of caregiving partners were female and the mean age was 50.7 years. Two thirds of partners, and over half of persons with SCI were in paid employment and the majority of couples had formed their relationship after SCI had occurred. More than 30% of participants reported financial strain, mean net equivalence household income per month was over 4300 CHF, participants had been in formal educations for on average 14 years, and rated themselves with a subjective social position of around six on a scale from one to ten. On average, the partners provided 2 hours a day of care for the person with SCI, but reported an average score of 7.4 on subjective caregiver burden.

**Table 1:** Basic characteristics of the pro-WELL baseline population

	Caregiving partners	Persons with SCI
Total [Missing values: SCI; partner]		
**Sociodemographic characteristics**		
Continuous	Mean (SD)	Mean (SD)
Age (in years)	50.7 (10.0)	51.7 (9.4)
Net-equivalent household income (in CHF) [17;18]	4333 (1560)	4495 (1473)
Years of formal education [7;2]	14.0 (3.2)	13.8 (3.2)
Subjective social position (range 0–10) [5;4]	6.0 (1.6)	5.8 (1.8)
Duration of caregiving (in years) [7]	18.8 (11.0)	–
Categorical	n (%, 95 CI)	n (%, 95 CI)
Female [0;0]	86 (72.9, 64.0–80.2)	36 (27.1, 19.8–35.9)
Financial strain [5;4]	39 (34.5, 26.2–43.9)	39 (34.5, 26.2–43.9)
Home ownership [2;7]	79 (66.4, 58.2–74.6)	81 (72.9, 63.8–80.5)
Paid employment [0;0]	76 (53.3, 44.2–62.3)	63 (64.4, 55.2–72.6)
**Lesion characteristics of the care-receiver**	n (%, 95 CI)	n (%, 95 CI)
Lesion severity [2]		
Incomplete paraplegia	–	36 (31.0, 23.2–40.1)
Complete paraplegia	–	45 (38.8, 30.3–48.1)
Incomplete tetraplegia	–	22 (19.0, 12.7–27.3)
Complete tetraplegia	–	13 (11.2, 6.6–18.5)
Aetiology [3]		
Traumatic	–	97 (84.3, 76.4–90.0)
Non-traumatic	–	18 (15.7, 10.0–23.6)
**Health status**	Mean (SD)	Mean (SD)
Secondary health conditions (SCI-SCS, range 0–24) [0]	–	10.8 (5.3)
Comorbidities (SCQ, range 0–5) [0;0]	0.8 (1.1)	0.9 (1.0)
**Psychosocial resources**	Mean (SD)	Mean (SD)
Quality of relationship (QRI, range 0–24) [3;2]	20.3 (3.6)	21.0 (2.9)
Loneliness (UCLA-SF, range 0–6) [3;2]	0.7 (1.3)	1.1 (1.5)
Self-efficacy [3;4]	15.5 (2.4)	15.0 (2.7)
**Unfulfilled support needs**	n (%, 95 CI)	
Wish for more support in caregiving (yes) [16]	19 (16.2, 9.5–23.0)	–
**Caregiver burden**	Mean (SD)	
Objective (duration of daily care in hours) [12]	2.0 (3.5)	–
Subjective (ZBI-S, range 0–48) [5]	7.4 (7.2)	–

Abbreviations: *CHF* Swiss Francs, *CI* Confidence Interval, *QRI* Quality of Relationship Inventory, *SCI* Spinal Cord Injury, *SCI-SCS* Spinal Cord Injury-Secondary Conditions Scale, *SCQ* Self-Administered Comorbidity Questionnaire, *SD* Standard Deviation, *UCLA-SF* UCLA Loneliness Scale-short form, *ZBI-S* Zarit Burden Interview-short form

Weak and moderate within-person correlations demonstrated the uniqueness of different dimensions of SEP. High within-dyad correlations were only found for income and home ownership, demonstrating the lack of coherence within couples of more proximal measures of SEP such as financial strain or subjective social position (Additional file 1: Tables S1 and S2).

### Study aim 1: SEP and caregiver burden (actor effects)

Results provide tentative support for a negative association between SEP and caregiver burden. More specifically, caregiving partners with higher incomes, educational level, and subjective social position, and those who owned their own home or who indicated no financial strain reported a lower propensity for both subjective and objective caregiver burden. Education demonstrated a negative association with objective caregiver burden (OR 0.88, 95% CI 0.73–1.00; *p-value 0.05*), as did subjective social position with subjective caregiver burden (OR 0.66, 95% CI 0.48–0.92; *p-value 0.01*). For each additional year of formal education, caregivers reported a 12% lower risk of high objective burden, and for each rang up the ladder of subjective social position, a 34% lower risk of high subjective burden. Moreover, caregivers who owned a home had reduced odds for high subjective burden and caregivers who indicated financial strain had higher odds for high objective caregiver burden (Table 2).

**Table 2:** Actor and partner effects of socioeconomic position on subjective and objective caregiver burden: Unadjusted and adjusted odds ratios (OR) and their 95% confidence intervals (CI) from logistic regressions

	High subjective caregiver burden (ZBI-s ≥ 12)		High objective caregiver burden (≥3 h of daily caregiving)	
	Model 1	Model 2	Model 1	Model 2
	OR (95% CI)	OR (95% CI)	OR (95% CI)	OR (95% CI)
**Actor effects**				
Net-equivalent household income				
Per CHF 1000	0.93 (0.69–1.26)	0.94 (0.66–1.34)	0.96 (0.71–1.29)	0.88 (0.61–1.28)
Years of formal education				
Per year	0.89 (0.74–0.98)*	0.94 (0.79–1.12)	0.84 (0.71–0.99)*	0.88 (0.73–1.00)*
Subjective social position				
0–10	0.71 (0.52–0.96)*	0.66 (0.48–0.92)*	0.92 (0.70–1.20)	0.90 (0.67–1.22)
Financial strain				
No	Reference	Reference	Reference	Reference
Yes	2.08 (0.81–5.31)	2.34 (0.86–6.39)	2.20 (1.01–4.80)*	2.51 (1.05–6.02)*
Home ownership				
No	Reference	Reference	Reference	Reference
Yes	0.26 (0.10–0.67)**	0.17 (0.05–0.53)**	1.11 (0.43–2.85)	0.87 (0.29–2.62)
**Partner effects**				
Net-equivalent household income				
Per CHF 1000	0.84 (0.61–1.15)	0.87 (0.59–1.27)	0.99 (0.76–1.30)	1.15 (0.82–1.62)
Years of formal education				
Per year	0.92 (0.79–1.07)	0.96 (0.81–1.14)	0.82 (0.71–0.94)**	0.84 (0.72–0.97)*
Subjective social position				
0–10	0.87 (0.67–1.12)	1.00 (0.73–1.38)	0.89 (0.72–1.10)	1.20 (0.90–1.59)
Financial strain				
No	Reference	Reference	Reference	Reference
Yes	1.23 (0.46–3.23)	0.82 (0.28–2.42)	1.84 (0.84–4.04)	0.99 (0.40–2.49)
Home ownership				
No	Reference	Reference	Reference	Reference
Yes	0.26 (0.10–0.67)**	0.19 (0.06–0.58)**	0.98 (0.44–2.17)	0.91 (0.34–2.47)

Model 1: Unadjusted; Model 2: Adjusted for age, sex, employment status and lesion characteristics of partner with SCI.

Abbreviations: *CHF* Swiss Francs, *ZBI-s* Zarit Burden Interview-short form

*p<0.05; **p<0.01

### Study aim 2: the partners’ SEP and caregiver burden (partner effects)

Results for partner effects were less consistent as those for actor effects, although all unadjusted models demonstrated associations in the expected direction, i.e., lower objective and subjective caregiver burden in higher SEP groups. However, the direction of associations was reversed in some cases after adjustment. The education of the care-receiving partner and home ownership were consistently negatively associated with both, the subjective and the objective caregiver burden. In particular, with each additional year of education of the care-receiver, the odds of high objective caregiver burden was reduced (OR 0.84, 95% CI 0.72–0.97; *p-value 0.02*), even in adjusted analysis (Table 2).

### Study aim 3: potential mediators between SEP and caregiver burden

**Fig. 2:**
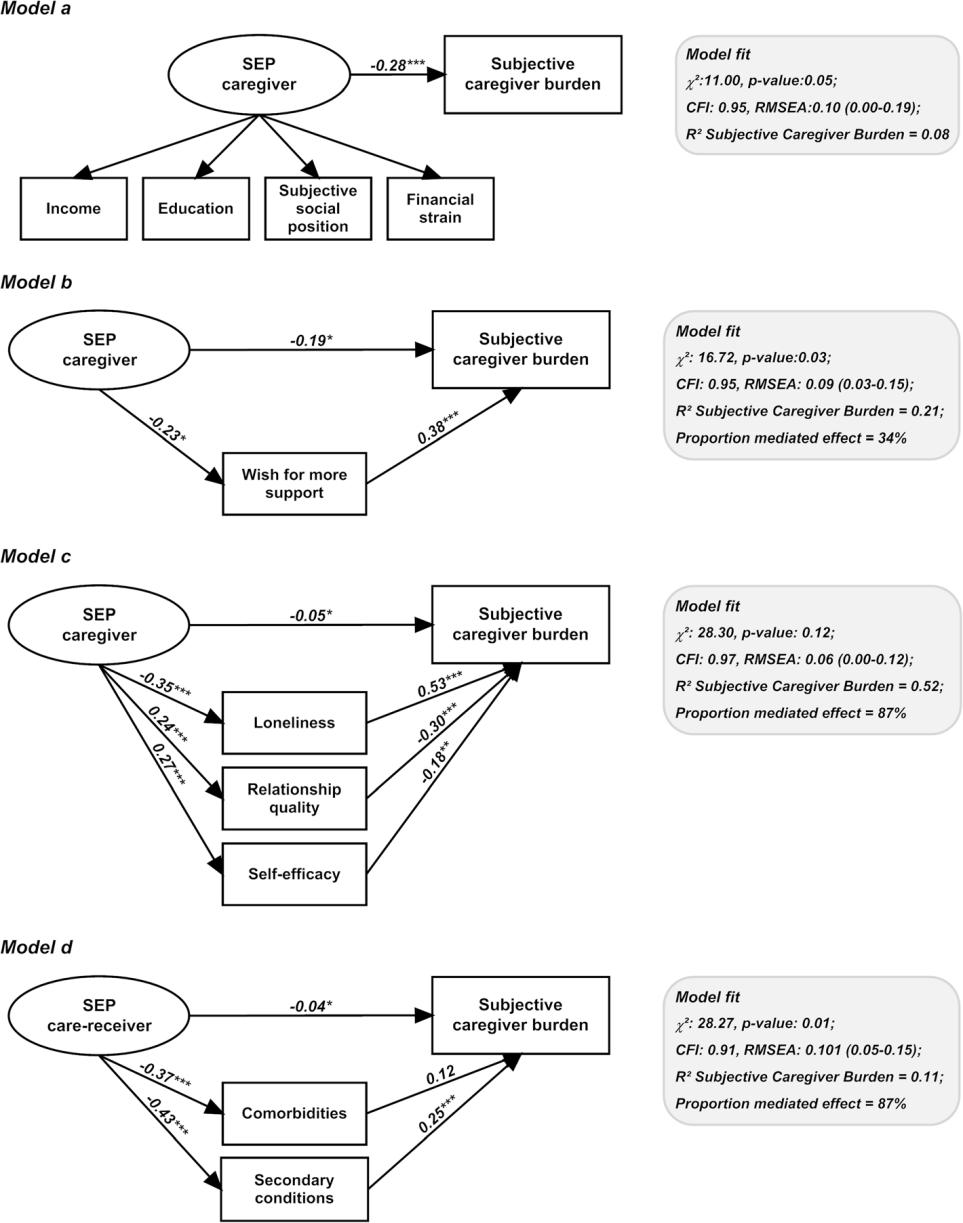
**a** Unmediated model. **b** Mediated by unfulfilled support needs of caregiver (parametrization of latent construct ‘SEP’ as in 2a). **c** Mediated by psychosocial resources of caregiver (parametrization of latent construct ‘SEP’ as in 2a). **d** Mediated by health status of care-receiver (parametrization of latent construct ‘SEP’ as in 2a)

Given that there were no significant associations between any of the potential mediators and objective caregiver burden (results not shown), mediation models were only run for the outcome subjective burden. The mediators included unfilled support needs, as represented by the variable wish for more support (Fig. 2b), psychosocial resources, as represented by relationship quality, loneliness and self-efficacy (Fig. 2c), and health status of the care-receiver, as represented by the comorbidities and secondary conditions of the person with SCI (Fig. 2d).

All models identified significant mediation effects (Table 3), which presents the indirect effect point estimates, and the bias corrected and accelerated CIs. Operative mediation was supported for all studied mediator groups (all 95% CIs excluding “0”, *p*-value < 0.05).

**Table 3:** Mediation effects of socioeconomic position on subjective caregiver burden through unfulfilled support needs, psychosocial resources and health status of care-receiver

	Point estimate (Unstandardized)	Product of coefficients		Bias corrected and accelerated 95% CI	
		SE	Z	Lower	Upper
Psychosocial resources	−1.02	0.33	−3.04	−1.56	−0.29
Unfulfilled support needs	−0.43	0.24	−1.77	−1.09	−0.07
Health status of care-receiver	−0.67	0.42	−1.58	−1.42	−0.37

Abbreviations: *CI* Confidence Interval, *SE* Standard Error

## Discussion

This is – to the best of our knowledge – the first study to investigate the association of SEP with both objective and subjective caregiver burden using a wide range of different SEP indicators, and applying a dyadic perspective. Our study findings support the hypothesis that low SEP is associated with increased caregiver burden, and also highlight the role of both the caregivers’ and the care-receivers’ social standing in shaping the caregiver experience. More specifically, we identified the education of caregivers and care-receivers as important determinants of objective caregiver burden and that more proximal measures of SEP, such as subjective social position and financial strain, were related to both subjective and objective caregiver burden. Furthermore, our findings confirm the hypothesis that unfilled support needs, low availability of psychosocial resources, and the poor health status of the care-receiver mediated the association between SEP and subjective caregiver burden. However, we found no evidence for mediation effects in the association between SEP and objective caregiver burden.

### SEP and caregiver burden

We identified the educational attainment of both the caregiver and care-receiver as the most robust determinant of objective burden. This finding is in line with previous studies that demonstrated a higher level of caregiving intensity in those with lower educational attainment [7, 9, 54, 55]. Since educational status remains relatively stable over the adult life course, education level may reflect an intrinsic resource that captures the capability of caregivers to manage caregiving tasks efficiently and effectively. Our results also suggest that care-receivers with higher educational attainment have superior skills in managing their health, appraising health information, accessing necessary formal support and communicating with health professionals, and as a result require less intensive caregiving from their partners [56, 57]. Care-receivers with a higher education may also be more able to cope psychologically with their condition and thereby reduce the burden of care for their caregiving partners [57].

Proximal measures of SEP, such as subjective social position and financial strain, were related to both subjective and objective caregiver burden. However, the directionality of the association is here more difficult to distinguish than in the case of education, given that these indicators of SEP are subjective constructs, which may be impacted by multiple external factors including the caregiver role itself. For example, the burden of caregiving may restrict the individuals’ ability to participate in social activities and in the labour market, influencing the subjective feeling of one’s social status and their earning capacity. Moreover, the additional costs of disability, such as costs for external services, assistive devices or adaptive technologies would likely impact upon the financial strain of both the caregiver and care-receiver, especially in those with more severe disabilities requiring more informal caregiving. In contrast, it may also be the case that those with limited financial resources are less able to access services and devices, thereby increasing the burden of care for those who are most socially disadvantaged. Much of the research addressing social inequalities in caregiving has explored the role which SEP has on the adoption of the caregiver role. Such research has found that it is not necessarily the case that only those with little paid employment take on caregiving, but that those who do take on the caregiver role reduce their hours of paid employment and look for more flexible working arrangements, often resulting in a reduction in salary [58]. In this sense caregiving intensity would have a direct effect on SEP and perceptions of financial strain; furthermore the modification of job role may impact upon the perception of subjective social position. Home ownership was also seen to be strongly associated with reduced subjective caregiver burden. In comparison to income, which is an indicator of monthly earnings, home ownership is an indicator of household wealth [59]. Wealth could be interpreted as a measure of financial stability and has been linked to multiple improved health outcomes [60]. Improved health status in care-receivers may lead to a reduced demand for informal care and to reduced stress for caregivers.

### Mediators on the pathway from SEP to caregiver burden

This study found evidence for the mediation effects of several material and psycho-social resources which are often unequally distributed within society. In terms of material resources we explored the effect of unfulfilled support needs, and used this as a proxy for lack of access to, and dissatisfaction with, health and support services. The importance of unmet emotional and medical needs in predicting caregiver burden has been identified in previous research, however, the social distribution of these unmet needs has not been documented yet [61]. This is also the case for other potential mediators under study, including self-efficacy and social relationships [13, 14]. Our research supports the assumption that the disparities in the caregiving experience are attributable to the social gradient of disability, as we demonstrated that persons with SCI with a lower SEP were more vulnerable to poor health and therefore require more intensive care from their partners. This supports previous research indicating that working class individuals were more likely to provide care for a spouse because of the higher prevalence of disability among this group [19].

### Implications

Our findings indicate that caregivers with a low SEP are at higher risk for exposure to caregiver burden and should therefore receive special attention when planning interventions to reduce the burden of care. The mediation analysis highlighted potential modifiable factors which could be targeted by appropriate interventions. For instance, specific training for socially disadvantaged caregivers may include guidance on how to access information and services, and strategies for coping with stressful situations. Moreover, persons with SCI with lower educational levels should be supported by health professionals in their health management, as strengthening the ability to self-manage the disability may reduce the burden of care for their spousal caregivers. The dyadic nature of the study has also highlighted the importance of considering the couple as a unit in the targeting of interventions aimed at reducing caregiver burden [62]. This study also highlights the need for a prospective, longitudinal study that dynamically investigates SEP and caregiver burden over the life course in SCI, from date of injury onwards.

### Strengths and limitations

This is the first study in couples coping with disability that takes into account a broad range of different indicators of SEP from both the caregiver and the care-receiver to link it with two conceptually distinct dimensions of the caregiver burden. Using this dyadic approach, the SEP of both persons can be considered as potentially influential, particularly as we found the reported subjective social position and their education to be weakly associated within dyads. Validated measures for most of the constructs under study were used and data were collected with high quality standards. We identified potential confounders by elaborating DAGs, and accounted analysis for potential item-nonresponse bias. Further, the pro-WELL study was nested within a large cohort study and socio-demographic and lesion characteristics of persons with SCI were well represented compared to the source population [29].

Due to the lack of longitudinal data for this specific research question, we were unable to test for causality, and it is presumed that many of the relationships under study may be bidirectional in nature, in particular those concerning the more subjective evaluations of SEP and those in relation to income as described above. The small sample size of this study prevented a more complex data analysis, as for example by not allowing the addition of confounders in the structural equation models. The limited sample size has also diminished the statistical power of analysis [63], calling for cautious interpretation of results. A further limitation of our study is the self-report nature of data and unmeasured confounding due to reporting bias. For example, we observed rather low levels of subjective caregiver burden and cannot exclude that social desirability issues biased this information, e.g. that burden was understated as caregiving partners did not want to put guilt on care-receiving partners. Moreover, we encountered high levels of missing data for income variables and were unable to evaluate potential subjective bias in the reporting of household income. Also, selection bias cannot be excluded as those with a higher caregiver burden felt unable to participate in the study due to the emotional and time related demands of caregiving.

## Conclusion

Our study in the context of SCI has provided dual support for the contextual role of SEP in the Stress Process Model of caregiving. Strengthening the psychological resources of, and improving the access to support services for caregivers emerged as promising intervention targets for individual level policy programs that seek to reduce the subjective caregiver burden in couples of low SEP. Moreover, the objective burden of care might be reduced by supporting caregivers and care-receivers with lower educational level to better self-manage the disability. However, in light of potential biases due to self-report and sample selection, more evidence on the studied associations is needed to underpin policy implications.

## Supplementary information

**Additional file 1: Tables S1.** Within-person correlations of indicators for socioeconomic position. **Table S2.** Between-person correlations of indicators for socioeconomic position.

## Data Availability

The datasets used and/or analysed during the current study are available from the corresponding author on reasonable request.

## Abbreviations

CHF: Swiss Francs
CI: Confidence Interval
DAG: Directed Acyclic Graph
EKNZ: Ethical Committee of Northwest and Central Switzerland
OECD: Organisation for Economic Co-operation and Development
QRI: Quality of Relationships Inventory
SCI: Spinal Cord Injury
SCI-SCS: Spinal Cord Injury-Secondary Conditions Scale
SCQ: Self-Administered Comorbidity Questionnaire
SD: Standard Deviation
SEP: Socioeconomic Position
SwiSCI: Swiss Spinal Cord Injury Cohort Study
UCLA-SF: UCLA Loneliness Scale-short form
ZBI-S: Zarit Burden Interview-short form

## References

[1] Pickard L, Wittenberg R, Comas-Herrera A, Davies B, Darton R (2000). Relying on informal care in the new century? Informal care for elderly people in England to 2031. Ageing Soc.

[2] UNECE. UNECE Policy Brief on Ageing No. 22: The challenging roles of informal carers. UNECE Working Group on Ageing 2019.

[3] OECD. Help wanted? Providing and Paying for Long-Term Care. Paris: OECD; 2011.

[4] Federal Office for Public Health: Support for relatives providing care and nursing https://www.bag.admin.ch/bag/en/home/strategie-und-politik/politische-auftraege-und-aktionsplaene/aktionsplan-pflegende-angehoerige.html. Accessed 1 Nov 2019.

[5] Montgomery RJ, Gonyea JG, Hooyman NR (1985). Caregiving and the experience of subjective and objective burden. Fam Relat.

[6] Hirst M (2003). Caring-related inequalities in psychological distress in Britain during the 1990s. J Public Health.

[7] Saito T, Kondo N, Shiba K, Murata C, Kondo K (2018). Income-based inequalities in caregiving time and depressive symptoms among older family caregivers under the Japanese long-term care insurance system: a cross-sectional analysis. PLoS One.

[8] Wakabayashi C, Donato KM (2006). Does caregiving increase poverty among women in later life? Evidence from the health and retirement survey. J Health Soc Behav.

[9] Nabors N, Seacat J, Rosenthal M (2002). Predictors of caregiver burden following traumatic brain injury. Brain Inj.

[10] Ghosh S, Choi WY, Williams A, Duggleby W, Ploeg J, Markle-Reid M, Peacock S. Burden on caregivers of adults with multiple chronic conditions: Intersectionality of age, gender, education level, employment status, and impact on social life. Canad J Aging/La Revue canadienne du vieillissement. 2019:1–12. 10.1017/S071498081900045X 31475644

[11] Pearlin L, Mullan J, Semple S, Skaff M (1990). Caregiving and the stress process: an overview of concepts and their measures. Gerontologist.

[12] Bartley M. Health inequality: an introduction to concepts, theories and methods. Cambridge: Wiley; 2016.

[13] Kim H, Chang M, Rose K, Kim S (2012). Predictors of caregiver burden in caregivers of individuals with dementia. J Adv Nurs.

[14] Vitaliano PP, Russo J, Young HM, Teri L, Maiuro RD (1991). Predictors of burden in spouse caregivers of individuals with Alzheimer's disease. Psychol Aging.

[15] Campbell P, Wright J, Oyebode J, Job D, Crome P, Bentham P, Jones L, Lendon C (2008). Determinants of burden in those who care for someone with dementia. Int J Geriatr Psychiatry.

[16] Pot A, Deeg D, Van Dyck R (2000). Psychological distress of caregivers: moderator effects of caregiver resources?. Patient Educ Couns.

[17] Pinquart M, Sörensen S (2007). Correlates of physical health of informal caregivers: a meta-analysis. J Gerontol Ser B Psychol Sci Soc Sci.

[18] Williams A, Sethi B, Duggleby W, Ploeg J, Markle-Reid M, Peacock S, Ghosh S (2016). A Canadian qualitative study exploring the diversity of the experience of family caregivers of older adults with multiple chronic conditions using a social location perspective. Int J Equity Health.

[19] Glaser K, Grundy E (2002). Class, caring and disability: evidence from the British retirement survey. Ageing Soc.

[20] Minkler M, Fuller-Thomson E, Guralnik JM (2006). Gradient of disability across the socioeconomic spectrum in the United States. N Engl J Med.

[21] Reinhardt JD, Von Elm E, Fekete C, Siegrist J (2012). Social inequalities of functioning and perceived health in Switzerland–a representative cross-sectional analysis. PloS one.

[22] Fekete C, Siegrist J, Reinhardt JD, Brinkhof MW, Group SS (2014). Is financial hardship associated with reduced health in disability? The case of spinal cord injury in Switzerland. PloS one.

[23] Sacker A, Head J, Gimeno D, Bartley M (2009). Social inequality in physical and mental health comorbidity dynamics. Psychosom Med.

[24] Aarts MJ, Kamphuis CB, Louwman MJ, Coebergh JWW, Mackenbach JP, van Lenthe FJ (2013). Educational inequalities in cancer survival: a role for comorbidities and health behaviours?. J Epidemiol Community Health.

[25] Bickenbach J, Officer A, Shakespeare T, von Groote P, WHO (2013). International perspectives on spional cord injury.

[26] Scholten EW, Kieftenbelt A, Hillebregt CF, Groot S, Ketelaar M, Visser-Meily JM, Post MW (2018). Provided support, caregiver burden and well-being in partners of persons with spinal cord injury 5 years after discharge from first inpatient rehabilitation. Spinal Cord.

[27] OECD/WHO. OECD Reviews of Health Systems. Switzerland. Geneva: OECD; 2011.

[28] OECD. Income inequality update: income inequality remains high in the face of weak recovery. Paris: OECD COPE Centre for Opportunity and Equality; 2016.

[29] Fekete C, Brinkhof MW, Tough H, Siegrist J (2017). Longitudinal study of social participation and well-being among persons with spinal cord injury and their partners (pro-WELL). BMJ Open.

[30] Brinkhof MW, Fekete C, Chamberlain JD, Post MW, Gemperli A (2016). Swiss national community survey of functioning after spinal cord injury: protocol, characteristics of participants and determinants of non-response. J Rehabil Med.

[31] Fekete C, Segerer W, Gemperli A, Brinkhof MW, Group SS (2015). Participation rates, response bias and response behaviours in the community survey of the Swiss spinal cord injury cohort study (SwiSCI). BMC Med Res Methodol.

[32] UNESCO (1997). International standard classification of education (ISCED).

[33] Hagenaars AK, de Vos K, Zaidi MA (1994). Poverty statistics in the late 1980s: research based on micro-data.

[34] Adler N, Stewart J (2007). The MacArthur scale of subjective social status.

[35] Zarit SH, Reever KE, Bach-Peterson J (1980). Relatives of the impaired elderly: correlates of feelings of burden. Gerontologist..

[36] Bédard M, Molloy DW, Squire L, Dubois S, Lever JA, O'Donnell M (2001). The Zarit burden interview. A new short version and screening version. Gerontologist.

[37] Kline Paul (2013). Handbook of Psychological Testing.

[38] Schreiner AS, Morimoto T, Arai Y, Zarit S (2006). Assessing family caregiver's mental health using a statistically derived cut-off score for the Zarit burden interview. Aging Ment Health.

[39] Stagg B, Larner AJ (2015). Zarit burden interview: pragmatic study in a dedicated cognitive function clinic. Prog Neurol Psychiatry.

[40] Reiner I, Beutel M, Skaletz C, Brahler E, Stobel-Richter Y (2012). Validating the German version of the quality of relationship inventory: confirming the three-factor structure and report of psychometric properties. PLoS One.

[41] Hughes ME, Waite LJ, Hawkley LC, Cacioppo JT (2004). A short scale for measuring loneliness in large surveys results from two population-based studies. Res Aging.

[42] Robinson-Whelen S, Taylor HB, Feltz M, Whelen M (2016). Loneliness among people with spinal cord injury: exploring the psychometric properties of the 3-item loneliness scale. Arch Phys Med Rehabil.

[43] Schwarzer R, Jerusalem M (1995). Generalized self-efficacy scale. Meas Health Psychol User’s Portfolio Causal Contr Beliefs.

[44] Sangha O, Stucki G, Liang MH, Fossel AH, Katz JN (2003). The self-administered comorbidity questionnaire: a new method to assess comorbidity for clinical and health services research. Arthritis Rheum.

[45] Kalpakjian CZ, Scelza WM, Forchheimer MB, Toussaint LL (2007). Preliminary reliability and validity of a spinal cord injury secondary conditions scale. J Spinal Cord Med.

[46] Textor J, Hardt J, Knuppel S (2011). DAGitty: a graphical tool for analyzing causal diagrams. Epidemiology..

[47] White IR, Royston P, Wood AM (2011). Multiple imputation using chained equations: issues and guidance for practice. Stat Med.

[48] Kenny DA, Kashy DA, Cook WL, Simpson J (2006). Dyadic data analysis - methodology in the social sciences.

[49] Cook W, Kenny D (2005). The actor–partner interdependence model: a model of bidirectional effects in developmental studies. Int J Behav Dev.

[50] Green MJ, Popham F (2019). Interpreting mutual adjustment for multiple indicators of socioeconomic position without committing mutual adjustment fallacies. BMC Public Health.

[51] Weston R, Gore PA, Chan F, Catalano D (2008). An introduction to using structural equation models in rehabilitation psychology. Rehabil Psychol.

[52] Preacher KJ, Hayes AF (2008). Asymptotic and resampling strategies for assessing and comparing indirect effects in multiple mediator models. Behav Res Methods.

[53] Enders CK, Bandalos DL (2001). The relative performance of full information maximum likelihood estimation for missing data in structural equation models. Struct Equ Modeling.

[54] van der Heide I, Wang J, Droomers M, Spreeuwenberg P, Rademakers J, Uiters E (2013). The relationship between health, education, and health literacy: results from the Dutch adult literacy and life skills survey. J Health Commun.

[55] Volandes AE, Paasche-Orlow MK (2007). Health literacy, health inequality and a just healthcare system. Am J Bioeth.

[56] Galobardes B, Shaw M, Lawlor DA, Lynch JW, Smith GD (2006). Indicators of socioeconomic position (part 1). J Epidemiol Community Health.

[57] Mandemakers JJ, Monden CW (2010). Does education buffer the impact of disability on psychological distress?. Soc Sci Med.

[58] Berecki-Gisolf J, Lucke J, Hockey R, Dobson A (2008). Transitions into informal caregiving and out of paid employment of women in their 50s. Soc Sci Med.

[59] Robert S, House JS (1996). SES differentials in health by age and alternative indicators of SES. J Aging Health.

[60] Pollack CE, Chideya S, Cubbin C, Williams B, Dekker M, Braveman P (2007). Should health studies measure wealth?: a systematic review. Am J Prev Med.

[61] Hughes TB, Black BS, Albert M, Gitlin LN, Johnson DM, Lyketsos CG, Samus QM (2014). Correlates of objective and subjective measures of caregiver burden among dementia caregivers: influence of unmet patient and caregiver dementia-related care needs. Int Psychogeriatr.

[62] Schulz R, Czaja SJ, Lustig A, Zdaniuk B, Martire LM, Perdomo D (2009). Improving the quality of life of caregivers of persons with spinal cord injury: a randomized controlled trial. Rehabil Psychol.

[63] Altman DG, Bland JM (1995). Absence of evidence is not evidence of absence. Br Med J.

