# Supplementary material for: Social inequalities in the burden of care: a dyadic analysis in the caregiving partners of persons with a physical disability
Source: Int J Equity Health. 2019 Dec 31;19:3. doi: 10.1186/s12939-019-1112-1 (PMC6938621; doi:10.1186/s12939-019-1112-1)
Supplement: Supplementary file 1 — Additional file 1: Tables S1. Within-person correlations of indicators for socioeconomic position. Table S2. Between-person correlations of indicators for socioeconomic position. [file 12939_2019_1112_MOESM1_ESM.docx]

**Table S1: Within-person correlations of indicators for socioeconomic position**

|  | Income | Education | Subjective social position | Financial strain | Home ownership |
| --- | --- | --- | --- | --- | --- |
| Income | - |  |  |  |  |
| Education | 0.24 | - |  |  |  |
| Subjective social position | 0.56*** | 0.35*** | - |  |  |
| Financial strain | -0.43*** | -0.29* | -0.63*** | - |  |
| Home ownership | 0.10 | 0.15 | 0.39*** | -0.31*** | - |

**Table S2: Between-person correlations of indicators for socioeconomic position**

|  | **Persons with spinal cord injury** | | | | |  |
| --- | --- | --- | --- | --- | --- | --- |
| **Caregiving partners** |  | Income | Education | Subjective social position | Financial strain | Home ownership |
|  | Income | 0.76*** | -0.13 | 0.13 | -0.24 |  |
|  | Education | -0.12 | 0.08 | 0.14 | -0.01 |  |
|  | Subjective social position | 0.64*** | 0.32 | 0.29 | -0.54*** |  |
|  | Financial strain | -0.48** | 0.38* | -0.41 | 0.53** |  |
|  | Home ownership | 0.09 | 0.04 | 0.15 | -0.15 | 0.87*** |
